# Autologous stem cell transplantation for post-transplant lymphoproliferative disorders after solid organ transplantation: a retrospective analysis from the Lymphoma Working Party of the EBMT

**DOI:** 10.1038/s41409-021-01270-5

**Published:** 2021-04-16

**Authors:** Toby A. Eyre, Sophie Caillard, Herve Finel, Ariane Boumendil, Jaimal Kothari, Heiner Zimmermann, Ralf Ulrich Trappe, Virginie De Wilde, Eleni Tholouli, Edward Kanfer, Angus Broom, Gandhi Damaj, Mario Bargetzi, Tomáš Kozák, Inken Hilgendorf, Charles Crawley, Tessa Kerre, Marek Trněný, Emmanuel Bachy, Stephen Robinson, Silvia Montoto

**Affiliations:** 1 Department of Haematology, Cancer and Haematology Centre, Churchill Hospital, Oxford University Hospitals NHS Foundation Trust, Oxford, UK; 2 Nephrology Transplantation Department, Strasbourg University Hospitals, Strasbourg, France; 3 Lymphoma Working Party EBMT, Paris, France; 4 Department of Hematology and Oncology, DIAKO Ev. Diakonie-Krankenhaus Bremen, Bremen, Germany; 5 Department of Internal Medicine II: Hematology and Oncology, University Medical Centre Schleswig-Holstein, Campus Kiel, Kiel, Germany; 6 Department of Hematology and Oncology, Charité-Universitätsmedizin Berlin, Berlin, Germany; 7 Erasmus Hospital, Route de Lennik 808, 1070 Bruxelles, Belgium; 8 Manchester Royal Infirmary, Oxford Road, Manchester, UK; 9 Department of Haematology, Hammersmith Hospital, London, UK; 10 Department of Haematology, Western General Hospital, Edinburgh, UK; 11 Department of Haematology, Hospital Center University of Caen, Caen, Normandy France; 12 Department of Haematology, Kantonsspital, Aarau, Switzerland; 13 Department of Haematology, Charles University Hospital, Prague, Czech Republic; 14 Department of Haematology, Jena University Hospital, Jena, Germany; 15 Department of Haematology, Addenbrookes Hospital, Cambridge, UK; 16 Department of Haematology, Ghent University Hospital, Ghent, Belgium; 17 Department of Haematology, Hospices Civils, Lyon, France; 18 BMT Unit, University Hospital Bristol NHS Foundation Trust, Bristol, UK; 19 Department of Haemato-oncology, St Bartholomew’s Hospital, Barts Health NHS Trust, London, UK

**Keywords:** Non-hodgkin lymphoma, Cancer therapy, Medical research

## Abstract

Published data describing the efficacy and safety of autologous stem-cell transplantation (autoSCT) in post-transplant lymphoproliferative disorders (PTLD) is limited to case reports. This is a retrospective analysis of 21 patients reported to the EBMT registry who received an autoSCT for PTLD post solid organ transplant (SOT). Median age at autoSCT was 47 (range: 22–71) years. The commonest SOTs were kidney (48%) and liver (24%). Commonest histologies included DLBCL-type PTLD (14/21) and plasmacytoma-like PTLD (3/21). Patients received a median of two lines of therapy (range: 1–4) pre-autoSCT. ECOG performance status pre-autoSCT was 0 in 14% and 1 in 86%. Remission status pre-autoSCT was CR 47% and PR 38%. BEAM conditioning was used in 57% and high-dose melphalan in 10%. The median follow-up post-autoSCT was 64 months for alive patients. 3-year PFS was 62% [95% confidence interval (CI) 44–87%] and 3-year OS was 61% [95% CI:43–86]. There were 12 deaths, including four related to autoSCT. 100-day non-relapse-mortality (NRM) was 14% and 1-year NRM was 24%. This study suggests that autoSCT, although feasible and with potential therapeutic activity, is associated with a high NRM, primarily driven by infectious toxicity. A multi-disciplinary approach, expert microbiological input and stringent patient selection are required to optimise outcomes.

## Introduction

Post-transplant lymphoproliferative disorders (PTLD) represent a clinical and histopathological spectrum of disease; from reactive to aggressive malignant phenomena occurring typically in the setting of immunosuppression associated with solid organ transplantation (SOT) [1]. Although PTLD can present with a variety of histological subtypes, the most common form of PTLD has a CD20-positive, B-cell monomorphic diffuse large B cell lymphoma (DLBCL)-like histology. As a result of the relative rarity of PTLD, large prospective clinical trials which enable clear clinical treatment pathways have been understandably scarce. Moreover, patients with PTLD have historically been excluded from prospective clinical trials studying novel therapies in aggressive B-cell lymphoma due to the associated underlying immunosuppression, impaired performance status (PS) at presentation and associated organ dysfunction. To date, the only prospective evidence to guide management is limited to phase II trials [2, 3] in the front-line setting.

High-dose chemotherapy with autologous stem cell transplantation (autoSCT) is considered the standard treatment approach for patients with relapsing or refractory (R/R) aggressive non-Hodgkin lymphoma and classical Hodgkin lymphoma (cHL) [4–6]. Outcomes are well described in these settings, with clear treatment pathways and prognostic factors having been established. In contrast, there are no prospective or retrospective data to guide decision making with regards to the potential utility and safety of autoSCT in patients with R/R PTLD. The evidence-based data is limited to a small number of case reports across various histologies [7–9]. In patients unresponsive to rituximab monotherapy, the safety and efficacy of treatment with the combination of rituximab, cyclophosphamide, doxorubicin, vincristine and prednisolone (R-CHOP) has been demonstrated in prospective phase II trials [2, 3, 10]. In a small case series from the pre-rituximab era, 9 patients with PTLD after SOT R/R after first-line CHOP chemotherapy were treated with CE (carboplatin and etoposide). Five of 9 achieved durable complete remissions without further treatment [11]. In patients that relapse following R-CHOP or who are refractory to this treatment, conventional platinum-based salvage approaches followed by consolidation with an autoSCT in chemotherapy responsive patients akin to treatment used in DLBCL [5, 12, 13] are sometimes employed with little evidence base. Particular attention should be paid to the potential toxicities of salvage chemotherapy [14] in relation to the underlying SOT. The effect of salvage chemotherapy and autoSCT on the function of the transplanted solid organ is also poorly understood.

To begin to address these key unanswered questions, the Lymphoma Working Party of the European Society for Blood and Marrow Transplantation (EBMT) conducted a retrospective study analysing the outcomes of patients with PTLD following a SOT who received an autoSCT, either in the front-line or relapsed setting, and were reported to the EBMT registry. To our knowledge, this is the only series of patients with PTLD treated with an autoSCT as consolidation treatment for whom detailed toxicity, engraftment and survival outcome is described.

## Materials and methods

The EBMT is a voluntary organisation comprising more than 600 transplant centres. Member centres submit at least minimal essential data (Med-A form) from consecutive patients to the lymphoma registry. We conducted an international, multicentre, retrospective EBMT registry study to describe the characteristics and outcomes of patients ≥18 years of age with a known diagnosis of PTLD following a SOT, for which they received an autoSCT at some point (front-line or at relapse) between 2001 and 2017. Thirteen transplant sites across the EBMT registry participated in this study. All histological subtypes were included. Patients who developed PTLD following allogenic SCT were excluded. Transplant centres with potential patients were contacted to obtain additional information (Med-B and Med-C forms). Informed consent was obtained locally according to the regulations applicable at the time of transplantation. After 1 January 2003, all EBMT centres have been required to obtain written informed consent before data registration.

Remission status at autoSCT was defined according to EBMT definitions: complete response (CR) was defined as the disappearance of tumour masses and disease-related symptoms; partial response (PR) was considered when measurable lesions decreased by ≥50%. Relapse was defined as the occurrence of new sites of disease following a CR lasting for ≥3 months, whereas it was defined as progressive disease (PD) when CR had not been achieved. Stable disease (SD) was defined as patients with neither CR, PR or PD. Follow-up monitoring of patients for relapse/PD post-transplant was conducted according to local centre protocol. Overall survival (OS) was defined as the time from autoSCT to death from any cause. Progression-free survival (PFS) was defined as the time from autoSCT until disease relapse/PD or death from any cause. Non-relapse mortality (NRM) included all causes of death occurring after autoSCT in the absence of relapse/PD. PFS and OS were both estimated by the Kaplan–Meier method [15].

The primary endpoint of the study was PFS. Secondary endpoints included OS, NRM, cumulative incidence of relapse and engraftment. Additional information was collected on management of immunosuppressive therapy pre- and post- autoSCT, and transplanted organ function following autoSCT. The patient characteristics that were collected included age, gender, type and reasons for SOT. PTLD characteristics collected included bulk disease (≥7.5 cm in any diameter) at PTLD diagnosis and number and site of involved extranodal sites. Epstein-Barr Virus (EBV) status and histopathological PTLD subtype was assessed by clinician review (TE) of written histopathology reports. EBV was considered positive if expression was noted by either in situ hybridisation for EBV‐encoded small RNA (EBER) or by Latent Membrane Protein 1 expression by immunohistochemistry. Data on the number of prior therapeutic lines, ECOG PS at autoSCT, PTLD remission status at autoSCT, autoSCT conditioning regimen and haematopoietic stem cell source were also collected. Statistical analysis of baseline characteristics was descriptive. Follow-up was censored at the most recent medical visit or death. The database was locked on December 2019 for analysis.

## Results

Twenty-one patients were included in the analysis. Patient characteristics are summarised in Table 1 and PTLD characteristics and treatment-related details are summarised in Table 2. The median age of the cohort was 45 (range 21–70) years at PTLD diagnosis and the median age at autoSCT was 47 (range 22–71) years. 15 patients (71%) were female. The reasons for the underlying SOT are listed in Table 3. The most common type of SOT was kidney followed by liver SOT. Four patients required a second SOT (renal *n* = 3; liver *n* = 1). Three of these SOTs were performed prior to PTLD diagnosis and also therefore prior to autoSCT. One patient with HELLP syndrome required an immediate second liver SOT due to a non-functioning first liver SOT. A second patient required a repeat kidney SOT following a prior kidney-pancreas SOT for diabetes. A third patient with endocardial fibroelastosis required a kidney SOT 20 years after a previous heart SOT. A single patient with cystic fibrosis received a lung SOT and developed chronic renal failure following PTLD and the autoSCT and required a kidney SOT 5 years post-autoSCT.

**Table 1:** Baseline patient characteristics.

Number	*N* (%)	Number available
Gender		21
Male	6 (29%)	
Female	15 (71%)	
Age at first solid organ transplant (SOT) [years (median, range)]	36 (4–55)	21
Time from SOT to diagnosis of PTLD [years (range)]	8.3 (0.2–23.7)	21
Age at PTLD diagnosis [years (median, range)]	45 (21–70)	21
Age at autoSCT [years (median, range)]	47 (22–71)	21
Number of SOT, number (%)		21
1	17 (81%)	
2	4 (19%)	
Type of SOT, number (%)		
Kidney	10 (48%)	21
Liver	5 (24%)	
Lung	2 (10%)	
Heart	2 (10%)	
Kidney-Pancreas	2 (10%)

**Table 2:** PTLD characteristics and treatment.

Number	*N* (%)	Number available
Bulk at diagnosis, number (%)		
Yes	5 (29%)	17
No	12 (71%)	
Histological PTLD subtype, number (%)		21
Monomorphic B-cell DLBCL-type	14 (67%)	
Plasmacytoma-like	3 (14%)	
Monomorphic B-cell Burkitt type	1 (5%)	
T-cell lymphoma	2 (10%)	
Polymorphic B-cell PTLD with plasmacytic differentiation	1 (5%)	
EBV expression in PTLD tissue at diagnosis^a^, number (%)		17
Yes	3 (18%)	
No	14 (82%)	
>1 Extranodal site, number (%)	4 (21%)	19
Time from diagnosis to autoSCT months [years (median, range)]	14 (4–89)	21
Number of lines of therapy before autoSCT (median, range)	2 (1–4)	21
Number of lines of therapy before autoSCT, number (%)		
1	5 (24%)	21
2	9 (43%)	
3	6 (28%)	
>3	1 (5%)	
Performance status at transplant (ECOG), number (%)		14
0	2 (14%)	
1	12 (86%)	
Stem cell source, number (%)		21
PBSCH	100%	
BM	0%	
Conditioning regimen for autoSCT, number (%)		
BEAM	12 (57%)	21
BEAM-R	1 (5%)	
BEAM-Zevalin	1 (5%)	
LEAM	1 (5%)	
LEAC	1 (5%)	
EAM	1 (5%)	
BCNU-Thiotepa	1 (5%)	
High Dose Melphalan	2 (10%)	
Other	1 (5%)	

*autoSCT* autologous stem cell transplantation, *PTLD* post-transplantation lymphoproliferative disease, *IPI* international prognostic index, *PTCL* peripheral T cell lymphoma, *ECOG* eastern co-operative oncology group, *PBSCH* peripheral blood stem cell harvest, *BM* bone marrow, *BEAM* carmustine, etoposide, cytarabine, melphalan, *LEAM* lomustine, etoposide, cytarabine, melphalan, *C* cyclophosphamide, *R* rituximab. *EBV status defined by LMP-1 immunohistochemistry or in situ hybridisation for EBV-encoded small RNA (EBER).

^a^EBV status defined by LMP-1 immunohistochemistry or in situ hybridisation for EBV-encoded small RNA (EBER).

**Table 3:** Reasons for solid organ transplantation.

Renal (n = 10)/Renal-Pancreas (*n* = 2)
Chronic glomerulonephritis (*n* = 4)
Diabetes-associated type 1 nephropathy (*n* = 2)
Vesiculo-ureteric reflux disease (*n* = 1)
Adult polycystic kidney disease (*n* = 1)
Uropathy not-otherwise specified (*n* = 1)
Renal failure not-otherwise specified (*n* = 1)
Multiple angiolipomas of the kidney in tuberous sclerosis (*n* = 1)
Focal segmental glomerulosclerosis (*n* = 1)
Liver (*n* = 5)
Acute steatosis, HELLP (*n* = 1)
Cirrhosis (Hepatitis C) (*n* = 2)
Cirrhosis (alcoholic liver disease) (*n* = 1)
Cirrhosis (alcohol and hepatitis C) (*n* = 1)
Other: lung (*n* = 2), cardiac (*n* = 2)
Cystic fibrosis (*n* = 2)
Congenital Cardiomyopathy Secondary to Endocardial Fibroelastosis (*n* = 1)
Not documented (*n* = 1)

The median time from initial SOT to first diagnosis of PTLD was 8.3 years (range 0.2–24). A variety of immunosuppressive therapeutic approaches were used, consistent with the timing, range of centres and SOT types studied. Steroids, calcineurin inhibitors, azathioprine and mycophenolate combinations were typically used as immunosuppressive treatment pre and post autoSCT (Table SI).

Histological PTLD subtypes included monomorphic B-cell DLBCL-type PTLD most commonly but also plasmacytoma-like, T-cell lymphoma PTLD, and monomorphic B cell Burkitt-type PTLD. EBV expression was evaluable in 17 patients. It was unknown or not tested the other 4 patients. EBV expression was positive in 3 (18%) of these 17 patients in whom their EBV tumour status was known. Expression in these 3 patients was either by EBER in situ hybridisation (*n* = 1) or LMP-1 immunohistochemistry (*n* = 2).

The median time from PTLD diagnosis to autoSCT was 14 months (4–89). Patients received a median of 2 treatment lines (range 1–4) prior to autoSCT. The timing of autoSCT differed according to histological subtype. Patients with monomorphic B-cell DLBCL-type PTLD received a median of 2 (range 1–3) prior lines of therapy, patients with plasmacytoma-like PTLD received a median of 1.5 (range 1–2) prior lines of therapy whereas patients with T-cell lymphoma PTLD received a median of 2.5 (range 2–3) prior lines pre-autoSCT. Remission status pre-autoSCT was as follows: CR 47%, PR 38%, SD 10%, PD 5%.

**Fig. 1:**
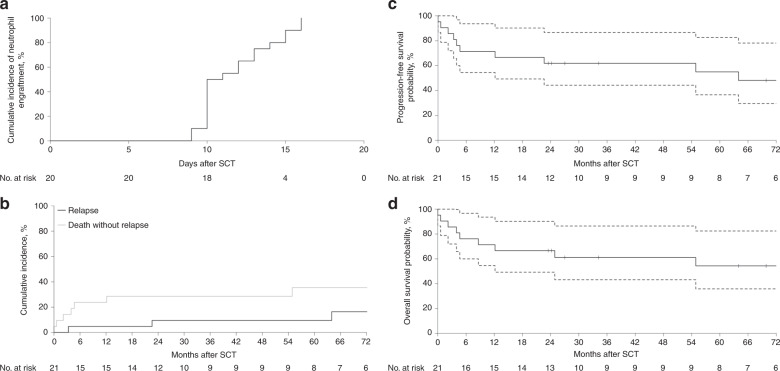
Survival, Relapse and Engraftment for PTLD autoSCT patients. **A** Cumulative incidence of neutrophil engraftment. **B** Cumulative incidence of relapse and death without relapse. **C** Progression-free survival with 95% confidence intervals. **D** Overall survival with 95% confidence intervals.

The most commonly used autoSCT conditioning regimen was carmustine, etoposide, cytarabine and melphalan (BEAM) followed by high dose melphalan. No patients received total body irradiation conditioning. Haematopoietic stem cell source was peripheral blood in all patients. Of the 16 patients with available data, 9 (56%) did not stop immunosuppression before autoSCT (1 relapsed and died, 5 died without relapse) and 7 (44%) patients did (1 relapsed, 3 died without relapse). Detailed data on post-autoSCT manipulation of immunosuppression was not available. Engraftment of neutrophils (defined as two consecutive absolute neutrophil counts ≥0.5 × 10^9/L) occurred in all 21 patients at a median of 10.5 (range: 9–16) days (Fig. 1A). Engraftment of platelets (defined as two consecutive unsupported platelet counts ≥20 × 10^9/L and no transfusion within 7 previous days) occurred in 17 of the 20 patients with data available with a median time of 13 (range 11–18) days.

The median follow-up post autoSCT was 64 months (range 23–127) for alive patients. Overall there were 12 deaths (Table SII) including four patients whose death was felt related to the autoSCT. One of these 4 deaths also occurred in the context of concurrent progressive PTLD but was definitely not due to PTLD. Infection, pulmonary dysfunction and multiorgan failure were predominant features in across this 12 patient cohort. The median time from autoSCT to death was 3 months (range, <1 month–9 months) for this group, whereas it was 40 months (range, 0–176) for the remaining 8 patients whose death was not attributed to autoSCT. The latest three deaths recorded occurred at 13, 14 and 15 years post autoSCT respectively. The causes of death in those three late events were unknown (*n* = 2) and infective endocarditis (*n* = 1). The 100-day NRM was 14% and the 1-year NRM was 24% (Fig. 1B). Table SIII summarises the 10 patients who died from non-relapse causes. Nine patients had received a single SOT (kidney *n* = 5, liver *n* = 2, lung *n* = 1, heart *n* = 1) and a single patient received a kidney-pancreas and then a subsequent repeat kidney SOT2  8 years following SOT1 - and 3 years prior to the autoSCT. The median time from latest SOT to autoSCT was 8.7 years (range 1.8–14.0 years). 8 of 9 patients with available data restarted some form of immunosuppression following the autoSCT.

Only 3 patients progressed (*n* = 1) or relapsed (*n* = 2) following autoSCT. Two of them died, one at 9 months post autoSCT of infection and another 25 months post- transplant of unknown cause (following a relapse at 23 months). Five patients (24%) received an autoSCT in the first line setting. All patients received chemotherapy prior to autoSCT (VCD *n* = 2, MATRix *n* = 1, Mega-CHOP *n* = 1 and R-CHOP *n* = 1) with no patients managed purely by reduction in immunosuppression. Two patients were in partial remission and three were in complete remission. Three of them died at 8, 55 and 176 months after autoSCT due to infection in the setting of progressive disease (PR1), unknown cause (PR1) and endocarditis (CR1), respectively. Two other patients obtaining CR1 were alive with no relapse at 34 and 120 months respectively. The 3-year PFS across the whole cohort was 62% [95% confidence interval (CI) 44–87%] and the 3-year OS was 61% [95% CI 43–86] (Fig. 1C–1D). At last follow-up when data were censored, 16 patients had a transplanted organ after the first SOT, 3 did not (all of which had a second SOT), and there were 2 patients for whom the functional status of the transplanted was unknown.

## Discussion

To the best of the authors knowledge, this EBMT case series represents the largest experience of autoSCT as a treatment modality for patients with PTLD. Until now, the use of autoSCT in patients with PTLD was based primarily on extrapolation of outcome data from the non-PTLD setting and on a very limited number of isolated case reports.

Whilst the survival outcomes require cautious interpretation in light of the small and heterogenous population evaluated, autoSCT as a consolidation strategy provided durable disease control in the majority of patients within this series, as only three relapses were recorded. We also demonstrate that this approach led to a 100-day NRM of 14% and a 1-year NRM of 24%. The toxicity associated with autoSCT in the setting of PTLD is considerably higher than the 2–5% range described post autoSCT in the setting of cHL [16], T cell lymphoma [17] or aggressive B-cell lymphoma [18, 19] in patients without a SOT and associated immunosuppression. This concerning finding is perhaps unsurprising but has never been previously demonstrated in the literature and has to be considered in the context of survivorship after SOT, with a 5-year survival ranging from around 50% for lung recipients to more than 90% for renal recipients. Evaluation of risk factors for infection such as dosing of co-existing immunosuppression, myelotoxicity of autoSCT conditioning, and pre-existent organ dysfunction should therefore be extremely carefully assessed when autoSCT in patients with PTLD is considered. The importance careful patient selection and of diligent multi-disciplinary communication including haematologists, physicians/surgeons managing the underlying SOT and microbiologists when managing toxicities here cannot be understated.

Our study has several limitations to acknowledge, which are inherent to the retrospective registry-based nature of the study, and the heterogeneity of types and treating centres in this small cohort. By definition, only patients from centres reporting to the EBMT and only cases recorded as PTLD were included. We cannot exclude the possibility that other patients have received an autoSCT for PTLD in Europe and have not been reported to the EBMT. The inherent difficulties of a registry study to deal with missing data also apply to this study. Both the baseline demographics (median age < 50 years, female predominance) and PTLD characteristics (rate of EBV-association 18%) of our patient cohort are unusual compared to previous trials and retrospective cohorts [2, 3, 20, 21], suggesting patient selection. Given the heterogeneous histological subtypes and the variable timing of autoSCT across the patients, it is challenging to provide generalizable recommendations. We also were not able to analyse in detail the function of the SOT pre or post autoSCT, but recognise this is of clinical relevance. Finally, as only patients who actually received an autoSCT are included in this study it is not possible to draw strong conclusions on the role of this approach in comparison with other strategies in the management of patients with PTLD, particularly as the denominator for those theoretically eligible for autoSCT is unknown.

An additional noteworthy finding following this EBMT project was the paucity of cases available for analysis. To overcome the possibility that other patients with PTLD have received an autoSCT in Europe and have not been reported to the EBMT registry, we contacted two European PTLD registries to ensure that we were not missing a significant number of patients. Ongoing collaborative efforts are required to ensure that patients with PTLD receiving intensive chemotherapy and autoSCT are collected within international registries. This will in turn enable considerably larger patient numbers to be studied over time in order to gain a greater understanding of more definitive survival, toxicity and prognostic factors.

In conclusion, we present outcomes of patients with PTLD treated with an autoSCT within the EBMT registry over the past two decades. We demonstrate that autoSCT, although feasible and with some evidence of therapeutic activity, is a significantly toxic procedure and a stringent selection of patients is necessary before recommending it. As such, multi-disciplinary team based patient care, expert microbiological input and careful patient selection are of utmost importance to optimise long term disease control and minimise toxicity. Ongoing international collaborative efforts are required to further the evidence in this field as well as the need for prospective international clinical trials.

## Supplementary information

Supplementary Tables
