# Supplementary material for: Autologous stem cell transplantation for post-transplant lymphoproliferative disorders after solid organ transplantation: a retrospective analysis from the Lymphoma Working Party of the EBMT
Source: Bone Marrow Transplant. 2021 Apr 16;56(9):2118–24. doi: 10.1038/s41409-021-01270-5 (PMC8410594; doi:10.1038/s41409-021-01270-5)
Supplement: Supplementary file 1 — Supplementary Tables [file 41409_2021_1270_MOESM1_ESM.docx]

**Table SI: Immunosuppression pre and post ASCT**

| Immunosuppressive therapy immediately prior to ASCT for PTLD post SOT | Number of patients | Number for which data available |
| --- | --- | --- |
| Tacrolimus  Prednisone  Cyclosporin  Azathioprine and Cyclosporin  Azathioprine and Tacrolimus  Cyclosporin, Mycophenolate and other  Cyclosporin and other  Mycophenolate and other  Mycophenolate and Tacrolimus  Tacrolimus and other  Other  None | 4  3  2  1  1  1  1  1  1  1  2  3 | 21 |
| Immunosuppressive therapy immediately after ASCT for PTLD post SOT | Number of patients | Number for which data available |
| Prednisone  Cyclosporin  Cyclosporin and other  Cyclosporin and Sirolimus  Mycophenolate and Cyclosporin  Mycophenolate and Sirolimus  Mycophenolate and Tacrolimus  Mycophenolate, Tacrolimus and Azathioprine  Tacrolimus and Prednisone  Other  None | 3  2  1  1  1  1  1  1  1  5  2 | 19 |

**Table SII: Causes of death**

| **Related to autoSCT (n=4)**   - Pulmonary bacterial infection and multiorgan failure (n=1)* - Bacterial infection, and multiorgan failure (n=1)* - Recurrent septic episodes, pneumonia and multiorgan failure alongside progressive disease (n=1) - Pulmonary infection (n=1)*   **Other (n=8)**   - Acute respiratory distress syndrome secondary to E coli bacteraemia (n=1)* - Bacterial infection (n=1)* - Renal failure (n=1)* - Infective endocarditis (n=1)* - Not documented/unknown (n=4; 3 without documented relapse*)   ***NRM n=10;** |
| --- |

**Table SIII: Summary of NRM cases (n=10)**

| Date of AutoSCT | Number of SOT | Date 1^st^ SOT | SOT1 | Reason SOT1 | Functioning SOT1 | Date SOT2 | 2nd SOT | Reason SOT2 | Functioning SOT2 | Immunosuppression pre-ASCT | Immunosuppression post ASCT | Immunosuppression stopped peri-auto-SCT? | relapse | Time to death (months) | Detailed cause of death |
| --- | --- | --- | --- | --- | --- | --- | --- | --- | --- | --- | --- | --- | --- | --- | --- |
| 2014-11-19 | 1 | 2002-07-15 | K | GN | Y |  |  |  |  | Prednisone | Other | N | N | 12.17 | **ARDS due to E coli** |
| 2013-01-02 | 1 | 2003-10-15 | Li | mixed cirrhosis, alcohol and hepatitis C | Y |  |  |  |  | Other | Other | N | N | 2.15 | **Bacterial infection, and MOF** |
| 2002-07-08 | 1 | 1988-06-25 | K | APKD | Y |  |  |  |  | Prednisone | Prednisone | Y | N | 4.67 | **Bacterial infection** |
| 2010-09-22 | 1 | 2002-07-15 | K | GN | NK |  |  |  |  | Other | Other | NK | N | 54.88 | **NK** |
| 2014-04-02 | 1 | 2012-02-25 | Lu | Cystic fibrosis | Y |  |  |  |  | Tacrolimus & Other | Tacrolimus & Prednisolone | N | N | 0.59 | **Pulmonary infection** |
| 2016-08-10 | 1 | 2003-03-01 | Li | hepatitis C | Y |  |  |  |  | None | None | Y | N | 0.03 | **Renal failure** |
| 2008-10-21 | 1 | 2007-01-15 | H | NK | NK |  |  |  |  | None | unknown | N | N | 3.95 | **Pulmonary bacterial infection and MOF** |
| 2001-04-05 | 1 | 1988-01-18 | K | chronic GN | N |  |  |  |  | Cyclosporin & Mycophenolate & Other | Cyclosporin | Y | N | 164.99 | **NK** |
| 2002-03-20 | 2 | 1991-12-12 | K-P | DM | N | 1999-11-09 | K | DM | Y | Cyclosporin | Cyclosporin & Sirolimus | NK | N | 152.20 | **NK** |
| 2004-03-18 | 1 | 2000-10-18 | K | Chronic GN | Y |  |  |  |  | Cyclosporin & Other | Prednisone | NK | N | 175.50 | **Infective endocarditis** |

Abbreviations: SOT: solid organ transplant, GN: glomerulonephritis, DM: diabetes mellitus, K: kidney, K-P: kidney-pancreas, Lu: Lung, Li: Liver, H: Heart, ARDS: acute respiratory distress syndrome, MOF: multiorgan failure, Y: yes, N: no, NK: not known
